# Additionality in Blue Carbon Ecosystems: Recommendations for a Universally Applicable Accounting Methodology

**DOI:** 10.1111/gcb.17559

**Published:** 2024-11-04

**Authors:** Alex Houston, Hilary Kennedy, William E. N. Austin

**Affiliations:** ^1^ School of Geography & Sustainable Development University of St Andrews St Andrews UK; ^2^ School of Ocean Sciences Bangor University Menai Bridge UK; ^3^ Scottish Association for Marine Science Dunstaffnage UK

**Keywords:** additionality, allochthonous, blue carbon, carbon crediting, IPCC, nature‐based solutions

## Abstract

Blue carbon ecosystems (BCEs) remove carbon dioxide from the atmosphere and store significant amounts of organic carbon (OC) in their soils. Consequently, the protection and restoration of BCEs may contribute to net greenhouse gas emissions abatement and help address the global challenges of both mitigating and adapting to climate change. An ongoing debate is whether OC sequestered out with the blue carbon (BC) project and transported to its present location (allochthonous) should be counted as ‘additional’. There are inconsistencies in the treatment of allochthonous carbon between BCE methodologies, potentially undermining the credibility of global BC accounting initiatives. To explore these inconsistences, we compare the methodologies which we were able to find online, with particular focus on the VERRA, IPCC and BlueCAM methodologies, and review the science underlying any approach to account for allochthonous OC. Our findings indicate that there are currently no robust scientific approaches to define an appropriate apportioning of allochthonous OC for discounting in the calculation of additionality. We therefore advocate for the inclusion of allochthonous OC in BC crediting projects when an observational and experimental approach does not support the calculation (and discounting) of the refractory allochthonous carbon contribution.

## Graphical abstract

The protection and restoration of blue carbon ecosystems may contribute to net greenhouse gas emissions abatement and help address the global challenges of both mitigating and adapting to climate change. An ongoing debate is whether organic carbon sequestered out with the blue carbon ecosystem project and transported to its present location (allochthonous) should be counted as ‘additional’. We advocate for the inclusion of allochthonous organic carbon in blue carbon ecosystem projects when an observational and experimental approach does not support the calculation of the refractory allochthonous carbon contribution.

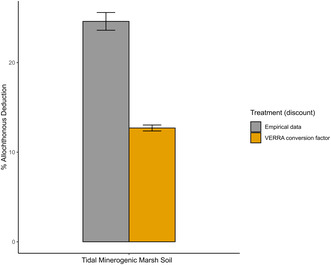

## 1 Introduction

Tidal marshes, seagrasses and mangroves are ‘actionable’ blue carbon ecosystems (BCEs) which globally store > 30,000 teragrams (Tg) organic carbon (OC) and could offset up to 3% of global emissions annually by 2030 through their protection and restoration (Macreadie et al. 2021). Other tidal wetlands, found across different continents, have very similar characteristics to the named actionable BCEs (Adame et al. 2024), and there is active interest in their inclusion for climate mitigation. In addition, there are those ecosystems that are defined as ‘emerging’ that have obvious potential to be deemed actionable, for example, unvegetated tidal flats (Howard et al. 2023). Given the large body of research surrounding tidal marshes, mangroves and seagrass meadows alongside their inclusion in regional, national and international policy, we focus only on these BCEs, while recognising that the issues around additionality will be relevant to all other BCEs. The potential for actionable BCEs to play a role in climate mitigation has enabled some governments (Herr and Landis 2016) to implement policies to adopt BCEs into their Nationally Determined Contributions (NDCs) to the 2016 Paris Climate Agreement (Howard et al. 2023) and formally include BCEs in national greenhouse gas (GHG) inventories (Crooks et al. 2018). The latest analysis of marine and coastal NDC commitments (Herr and Landis 2016) suggests a growing number of countries implementing the 2013 Intergovernmental Panel on Climate Change (IPCC) Wetlands Supplement (IPCC 2014; Lecerf et al. 2023), and some countries have developed national codes to facilitate voluntary carbon markets (VCM), such as BlueCAM in Australia, using the methodology that is consistent with IPCC guidance (Lovelock et al. 2022).

The interest in BCEs for their climate mitigation service is also manifest in the rapid demand for carbon credits by the private sector, with the restoration of BCEs projected to be worth > $10 billion from VCMs (Friess et al. 2022; Macreadie et al. 2021). The purchase of carbon credits allows entities to offset their carbon emissions through generation of additional carbon sequestration and/or avoided emissions from ecosystem protection and restoration or improved carbon management (e.g., reduction of methane emissions) (Friess et al. 2022).

To include BCE projects in VCMs, additionality must be demonstrated (Michaelowa et al. 2019). As defined in the Kyoto Protocol, additionality, in its widest terms, is ‘reductions in emissions that are additional to any that would occur in the absence of the certified project activity’ (UNFCC 1998). Determining the additional climate mitigation service of a project is crucial for ensuring genuine emissions reductions and/or avoided emissions (Michaelowa et al. 2019). Therefore, to demonstrate additionality, the net emissions in any project scenario must be lower than if the project or management activity had not been implemented (baseline scenario).

Globally, up to 67% of BCEs have been lost in recent centuries (Lotze et al. 2006). Tidal marshes have been lost at 0.28% per year since 2000 (Campbell et al. 2022), and mangroves have been lost at a rate of 0.1%–0.2% over a similar period and showed a marked reduction since pre‐2000 levels (Friess et al. 2020; Hamilton and Casey 2016). In the absence of reliable global‐scale mapping of seagrass habitats, there are no available calculations of global areal change, but for some sites, there is evidence of extensive long‐term habitat losses (Dunic et al. 2021). The ongoing losses and pressures on existing BCEs, as well as the high cost and complexity of restoring degraded BCEs, highlight the greater potential for BC additionality to be achieved through avoided losses of stored carbon rather than through restoration of BCEs (Howard et al. 2023). The protection of BCEs avoids the loss of irrecoverable carbon to the atmosphere (Goldstein et al. 2020), delivering immediate climate abatement through avoided emissions, whereas restored BCEs can take decades to achieve genuine emissions mitigation (Howard et al. 2023).

A full appreciation of carbon dynamics in BCEs requires an improvement in the scientific evidence base to determine how natural and anthropogenic disturbance contribute to changing rates of carbon dioxide emissions and removals and better constrain the role that BCEs play in the global carbon cycle (Macreadie et al. 2019; Santos et al. 2022; Williamson and Gattuso 2022). One issue when accounting for additionality is to consider how much of the OC accumulating in the soil is derived from the vegetation via above‐ and belowground production (autochthonous) and how much of the OC accumulating in the BCE soils was initially produced and sequestered outside the project boundary (herein and allochthonous) and whether or not any of this allochthonous OC should be counted as ‘additional’ (e.g., Houston, Garnett, and Austin 2024).

Soils of coastal mangroves, tidal marshes and some seagrass communities/ecotypes can be broadly divided into two types: those dominated by autochthonous inputs (typically organogenic soils) and those dominated by, for example, riverine inputs which tend to accumulate greater proportions (up to 90%) of allochthonous OC (often forming minerogenic soils) (Komada et al. 2022; Krause et al. 2022; Ricart et al. 2020; Xiong, Liao, and Wang 2018). Within the sub‐classification of BCEs with minerogenic soils, the amount of allochthonous OC accumulating in them varies significantly spatially, partially due to the geomorphic setting (Balke and Friess 2016). For example, mangroves in estuarine systems with a large tidal range have been shown to accumulate a greater amount of allochthonous OC than those in a microtidal setting (Balke and Friess 2016; Rovai and Twilley 2021). Other factors include the suspended sediment concentration and the density and height of the vegetation present which influences sediment deposition rates (Mueller et al. 2019; Rovai and Twilley 2021).

Allochthonous and autochthonous OC both contain different quantities and qualities (reactivity and stability) of OC, commonly referred to as labile and refractory (or recalcitrant) based on their perceived resistance to decomposition (Leorri et al. 2018). Labile carbon is defined as bioavailable and is expected to be remineralised over short timescales. Refractory carbon is assumed to be non‐reactive in most situations within centennial timescales (up to 100 years). Past research has found that autochthonous and allochthonous OC sources contain labile and refractory components, and that allochthonous sources usually contain a greater proportion of refractory carbon (Komada et al. 2022; Trevathan‐Tackett 2016; Van de Broek et al. 2018). The stability of the refractory allochthonous component is thought to be derived from either the chemical composition of the OC and its resistance to remineralisation or the association of soil OC with metals and fine‐grained minerals, which can physically and chemically stabilise the OC (Bianchi et al. 2024). Mineral associations have been found to enhance the residence time of allochthonous OC in BCE soils relative to autochthonous OC (Komada et al. 2022).

In certain cases, it has been contended that some, or all, of the allochthonous OC should be deducted from the overall pool of soil OC accumulating in BCE projects (ACR 2017; Gold Standard 2024; VERRA 2023). In the Australian Carbon Credit Unit scheme, however, it is argued that no allochthonous OC should be discounted (Lovelock et al. 2023). To further inform this debate, an improved understanding of OC reactivity and its relation to autochthonous/allochthonous provenance is required.

In this study, we review the treatment of allochthonous carbon in BCE methodologies, which we were able to find online, including VCM mechanisms (VERRA VM0033 Methodology for Tidal Wetland and Seagrass Restoration; Gold Standard Methodology for Sustainable Management of Mangroves), international policy frameworks (IPCC 2013 Supplement to the 2006 IPCC Guidelines for National Greenhouse Gas Inventories: Wetlands; Clean Development Mechanism Afforestation and Reforestation of Degraded Mangrove Habitats), and national and regional policy frameworks (BlueCAM Tidal Restoration of Blue Carbon Ecosystems Methodology Determination 2022, Australia; a methodology for seagrass carbon, France; a methodology for seagrass carbon, Japan; The Restoration of California Deltaic and Coastal Wetlands, USA; ACR 2017; Comte et al. 2024; Gold Standard 2024; IPCC 2014; Kuwae et al. 2022; Lovelock et al. 2022; UNFCC 2013; VERRA 2023). We argue that continuing with a mathematical approach to estimating the stability of allochthonous OC is unlikely to produce accurate carbon accounting, but that expanding analytical work on BCE soils affected by a range of management activities may result in a reduction of the uncertainty surrounding current methodologies and lead towards a more coherent treatment of allochthonous OC in BCE projects.

## 2 Comparison of the Treatment of Allochthonous OC in BCE Methodologies

Looking in more detail at the treatment of allochthonous OC in three methodologies, including the VERRA (VCM), IPCC (international policy) and BlueCAM (national policy) approaches, highlights that each method estimates a different source apportionment and underlying reactivity for soil OC (Table 1). This divergence in the treatment and accounting of allochthonous carbon is observed in the other available methodologies (Table S1), further highlighting the widespread inconsistencies in approach and treatment of additionality.

**TABLE 1:** Treatment of allochthonous OC by three example BCE carbon crediting methodologies: VERRA, BlueCAM and IPCC.

Methodology	Treatment of allochthonous OC	Reasoning
2013 Supplement to the 2006 IPCC Guidelines for National Greenhouse Gas Inventories: Wetlands Methodological Guidance on Lands with Wet and Drained Soils, and Constructed Wetlands for Wastewater Treatment (IPCC 2014)	Allochthonous carbon is not mentioned in this documentation	Pg 176: ‘The carbon stock is taken as all soil carbon except any refractory (unoxidisable) carbon. In mangrove soils, 4% of the carbon stock is refractory (Annex 4A.4) and this is taken to be representative of the refractory carbon in tidal marshes and seagrass meadows as well.’ Pg 343: ‘Refractory carbon…Soil carbon that does not get broken down and released as dissolved or gaseous CO_2_ (predominantly by microorganisms) within the time scale of the inventory.’
BlueCAM (Lovelock et al. 2022)	Pg 12: ‘BlueCAM does not include discounts for allochthonous carbon that is trapped in coastal wetlands and incorporated within soils.’	Pg 12: ‘While allochthonous carbon has been detected in coastal wetland soils in Australia, contributions of allochthonous carbon were typically small compared to autochthonous sources in saltmarshes and mangroves (Saintilan et al. 2013), but large in some sites for seagrass (Samper‐Villarreal et al. 2016).’ Pg 12: ‘In Australia's existing accounting framework, organic carbon that is eroded from landscapes and transported to the coast is assumed emitted as CO_2_, and therefore any portion of this organic carbon trapped in coastal wetlands could be considered an avoided emission (Kelleway et al. 2020)’. Pg 12: ‘Future development of BlueCAM could revisit the importance of allochthonous carbon sources and sinks.’
VM0033 Methodology for Tidal Wetland and Seagrass Restoration v2.1 (VERRA 2023)	Pg 38: ‘A deduction from the estimate of CO_2_ emissions from the SOC pool must be applied to account for the percentage of sequestration resulting from allochthonous soil organic carbon accumulation.’ Pg 38: ‘Estimation may be made for total or recalcitrant allochthonous carbon.’ Pg 39: ‘%Calloch may be estimated using either: (1) Published values (2) Field‐collected data (3) Modeling.’	Pg 56: ‘The determination of the deduction for allochthonous carbon is mandatory for the project scenario unless the project proponent is able to demonstrate that the allochthonous carbon would have been returned to the atmosphere in the form of carbon dioxide in the absence of the project.’ Pg 56: ‘If the organic surface layer exceeds 10 cm, the soil is deemed organic and no deduction is required.’

VERRA allows for the calculation of the refractory allochthonous fraction using field‐collected data for the project, or values from relevant literature (Table 1, Box 1). VERRA does not require any deduction from projects with organogenic soils, as the refractory allochthonous fraction, that is, the mineral‐associated organic matter (MAOM) fraction, is assumed to be minimal in these settings (VERRA 2023).

BOX 1 **VERRA Calculation of refractory allochthonous carbon using the Needelman et al. (2018) methodology applied to a wetland soil dataset (Mirabito and Chambers 2023).**

1.5% of the mineral mass of minerogenic soils is assumed to be refractory allochthonous carbon (Mayer 1994; Needelman et al. 2018; VERRA 2023). This default conversion factor is derived from data from continental shelf sediments (Mayer 1994) rather than recognised actionable BCEs (Needelman et al. 2018).

The proportion of the total sample which is refractory allochthonous carbon is then calculated by multiplying the proportion of the sample which is mineral mass (remaining mass after loss‐on‐ignition [LOI] to calculate sample OM content) by the default 1.5% value defined above.

The refractory allochthonous carbon fraction is divided by the total OC (TOC) fraction and multiplied by 100 to calculate the % refractory allochthonous carbon deduction, which should be applied to TOC accumulating in the project soils. This method results in the minerogenic soils, with the lowest OC having the greatest refractory allochthonous deduction, and applying this default value to any soil with an OC content of < 1.895% would result in 100% OC discounting.

**FIGURE 1:**
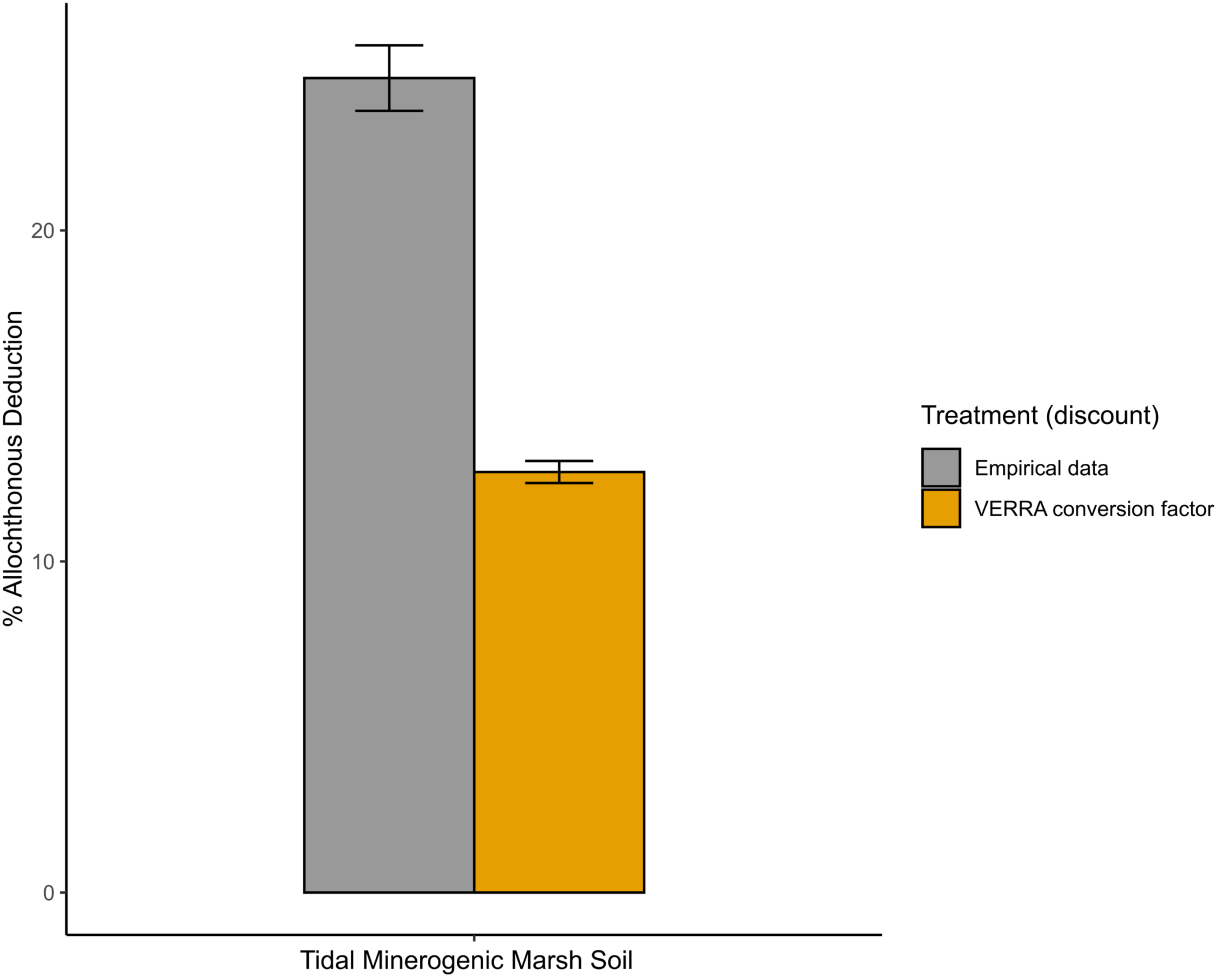
Allochthonous (%) carbon deductions estimated from %MAOM of a tidal minerogenic marsh soil (Mirabito and Chambers 2023) and the default VERRA deduction for refractory allochthonous carbon, represented as the MAOM fraction of the soil (Needelman et al. 2018).

Mirabito and Chambers (2023) estimated that in a minerogenic tidal marsh soil with OM 20.15 wt% and OC 9.4 wt%, 24.6 ± 0.99% of the measured OC was associated with MAOM. Applying the OM and OC contents of this site from Mirabito and Chambers (2023) using the VERRA method results in a refractory allochthonous deduction of 12.7 ± 0.33% of the OC (Figure 1).

While this site may not be typical of tidal marshes as a whole, it does suggest that further analyses are necessary to determine whether taking a percentage OC relative to mineral mass determined from LOI gives a representative and valid deduction to make. This has already been identified as a key science and policy question (Needelman et al. 2018). A clearer understanding of the extent and source of MAOM is needed, while further uncertainty arises from the lack of knowledge regarding the vulnerability of MAOM to destabilisation when the characteristics of the sedimentary or environmental setting are changed as a result of disturbance (de Nijs and Cammeraat 2020; Spivak et al. 2019).

Additionally, in the case of seagrass meadows found in tropical settings, CaCO_3_ can represent > 90% of the soil mass (Campbell et al. 2015). It is not known whether this mineral has any, or the same association with OM to immobilise OC as seen with allochthonous OC in other BCEs. Further research on environmental settings when either calcium carbonate or opal are prevalent in these soils is needed to fill this evidence gap.

In the IPCC GHG Inventory guidelines for coastal wetlands, a 4wt% discount for refractory OC is prescribed, regardless of autochthonous or allochthonous source (IPCC 2014. Table 1). The deduction is based on data from organogenic mangrove soils, where refractory OC is deemed resistant to remineralisation and reflects molecularly stable OC rather than MAOM. The IPCC methodology assumes the same proportion of refractory OC to be present in both organogenic and minerogenic soils in all three BCEs, when presumably the role of MAOM in stabilising soil OC can be very different.

Unlike either the VERRA and IPCC methodologies, the Australian BlueCAM methodology does not require a deduction of any OC as MAOM or remaining refractory OC (Lovelock et al. 2022; Table 1). The inclusion of all soil OC accumulating in the BCE project area as additional is justified by arguing that if the project did not exist, the OC would not have been deposited and would have remained unaccounted for in any existing GHG Inventory or NDCs (Lovelock et al. 2022, 2023; Table 1).

Of the other BC methodologies we found online (Table S1), the Methodology for Sustainable Management of Mangroves (Gold Standard 2024) and The Restoration of California Deltaic and Coastal Wetlands (ACR 2017) both advocate for the deduction of allochthonous carbon. However, the Gold Standard methodology allows for the inclusion of allochthonous carbon if it can be proven that it would be remineralised to the atmosphere in the absence of the BC project. Conversely, the Seagrass methodologies for neither Japan (Kuwae et al. 2022) nor France (Comte et al. 2024) mention the deduction of the allochthonous OC in their respective documentation (Table S1). The Clean Development Mechanism methodology for mangrove afforestation and reforestation also does not require a deduction for allochthonous carbon (Table S1; UNFCC 2013).

The BC methodologies considered in this study provide different justifications for the treatment of allochthonous OC in BCE projects (Table 1; Table S1). Table 2 applies the required allochthonous OC discount for the VERRA, IPCC and BlueCAM methodologies in Table 1 to an example dataset from Mirabito and Chambers (2023). This demonstrates the impact of the contradictory treatment of allochthonous OC between these methodologies on the amount of carbon deemed additional for a given BCE carbon crediting project.

**TABLE 2:** Allochthonous/refractory OC deductions required by three BCE carbon crediting methodologies for an example marsh soil dataset (Mirabito and Chambers 2023).

Methodology	Allochthonous/refractory OC deduction from OC stock (Mean ± Standard deviation)
IPCC—2013 Supplement to the 2006 IPCC Guidelines for National Greenhouse Gas Inventories: Wetlands Methodological Guidance on Lands with Wet and Drained Soils, and Constructed Wetlands for Wastewater Treatment	4%
BlueCAM	0%
VERRA—VM0033 Methodology for Tidal Wetland and Seagrass Restoration v2.1	12.7% ± 0.33% (conversion factor) OR 24.6% ± 0. 99% (empirical data)

In addition to the difference between the methodologies in the required deduction of refractory/allochthonous carbon (Table 2), there is a large difference in allochthonous carbon deduction for the VERRA methodology depending on whether empirical or default values are used for the example dataset of Mirabito and Chambers (2023) (Table 2). This demonstrates that the use of default conversion values instead of measured values can result in inaccurate/inappropriate allochthonous deductions.

## 3 Organic Carbon Reactivity and Its Relation to the Autochthonous/Allochthonous Source

Research on the post‐depositional turnover of OC in tidal marshes has determined that autochthonous OC is cycled at a faster rate than aged, allochthonous OC, and that the latter contributes disproportionately to long‐term storage (Komada et al. 2022; Leorri et al. 2018; Mueller et al. 2019; Van de Broek et al. 2018). Based on this evidence, it has been assumed that autochthonous OC generally contains a greater proportion of labile compounds that do not become associated with MAOM and the ‘protection’ it provides, whereas allochthonous carbon generally contains a greater proportion of refractory compounds and/or is ‘protected’ through associations with minerals (Komada et al. 2022).

Soil OC degradation differs between BCEs partly because its bioavailability depends more on the prevailing environmental conditions than the molecular reactivity of the OC itself (Luk et al. 2021; Noyce et al. 2023; Spivak et al. 2023). For example, stabilisation mechanisms such as mineral protection can be altered when exposed to different environmental conditions such as elevated oxygen availability (Spivak et al. 2019). Recent evidence has empirically shown that allochthonous OC can be remineralised from BCE soils under experimental degradation (i.e., aerobic oxidation) scenarios (Houston, Garnett, and Austin 2024). By contrast, Spivak et al. (2023) found no evidence for the increased remineralisation of mineral‐associated OM from tidal marsh soils when exposed to elevated oxygen availability or upon the addition of labile OM. It therefore remains unclear (i) whether the MAOM represented preservation of autochthonous and/or allochthonous OC; or (ii) whether mineral‐protected allochthonous OC is stable in BCE soils and becomes a source of GHG emissions to the atmosphere when exposed to greater oxygen availability, for example, during drainage of tidal marshes. However, other processes associated with BCE degradation such as nutrient enrichment and increased tidal flushing are known to destabilise OC and affect mineral protection (Spivak et al. 2019).

As discussed above, the fate of any fraction of OC through remineralisation is highly variable (Kothawala et al. 2021). Therefore, while mineral‐associated allochthonous OC may be stable in healthy BCE soils, this may not be the case when the ecosystem is degraded. Understanding the fate of OC under different environmental/management scenarios will therefore be important in accounting for allochthonous OC as additional in BCE accounting methodologies. In the case of tidal marsh habitats, for example, adjacent depositional environments such as intertidal mudflats may provide useful evidence to determine whether allochthonous OC is remineralised at different rates across adjacent depositional environments. In this situation, the tidal marsh would represent an actionable BCE under IPCC guidelines, while the adjacent mudflat would be non‐actionable, but equivalent to the sediment type that would fill available accommodation space in a typical managed realignment project (Howard et al. 2023). Further insight may also be gained from laboratory‐based incubation studies where different stresses such as elevated oxygen availability can be applied under controlled conditions (Houston, Garnett, and Austin 2024; McTigue, Walker, and Currin 2021; Spivak et al. 2023).

## 4 Double Counting of Allochthonous Carbon

In addition to uncertainties surrounding the reactivity of allochthonous OC, there is a separate issue of double counting (Kelleway et al. 2020). Allochthonous OC may have been mobilised by natural or anthropogenic processes and its remineralisation accounted for in the location of its disturbance or during transport to a BCE at the coast. If complete remineralisation of anthropogenically disturbed soil has not occurred before the allochthonous OC is captured in BCE's soils, it could be considered double counting. For example, if the change to agricultural soils from a management activity such as tillage is calculated via a stock change, the GHG emissions associated with remineralisation and erosion are combined and the assumption would be that all eroded soil is remineralised. The fate of eroded soil OC has been questioned, with estimates of post‐erosion remineralisation varying from < 5% to 100% (Kirkels, Cammeraat, and Kuhn 2014; Lal 2003). Understanding how and when to discount terrestrial soil OC which is trapped and accumulating in BCE soils remains problematic yet highlights an important climate service. Double counting is not stated as a reason for discounting allochthonous OC in any of the documentation we found online (Table 1; Table S1). For example, in Australia, carbon eroded from terrestrial project areas is assumed to be lost to the atmosphere so any allochthonous carbon accumulation in BCE projects is therefore not considered to represent double counting and is accounted for as additional (Lovelock et al. 2022).

## 5 Methods for Calculating Allochthonous Contributions to BCEs

Current standard methods for calculating allochthonous contributions to BCEs are mostly prohibitively complex/expensive (e.g., biomarkers) or lack the resolution (e.g., stable isotopes) to gain insight into the relative contributions of autochthonous and allochthonous carbon to most BCEs (Geraldi et al. 2019). There are other approaches that could facilitate calculation of allochthonous contributions, such as calculating the proportion of MAOM‐OC (Hamada et al. 2024). The role of MAOM in long‐term storage of OC in terrestrial soils has been documented (Hemingway et al. 2019), and there is some evidence for a similar role in BCE soils (Hamada et al. 2024; Komada et al. 2022), but the exact role of MAOM in the storage and remineralisation of OC in these systems remains unclear (Hamada et al. 2024; Spivak et al. 2019). Methods for calculating allochthonous contributions to BCEs will also need to resolve the proportion of accumulating and buried carbon which is vulnerable to loss to the atmosphere. Unfortunately, the required deductions for allochthonous OC in some accounting methodologies result in BCE projects using default factors derived from data from entirely different systems to estimate allochthonous deductions, with large discrepancies from empirical calculations of the refractory allochthonous fraction (Figure 1).

## 6 Financial Viability of BC Crediting Projects

A constraint to the undertaking of many BC projects is the cost of their implementation and monitoring against the value of the carbon credits/climate mitigation achieved (Howard et al. 2023). The discounting of allochthonous OC in the assessments of additionality may further challenge the viability of such projects based on carbon crediting investment calculations (e.g., Burden et al. 2023). For example, according to the VERRA default deduction factor, we estimate that BCE soils with < 1.895 wt% OC content (Box 1; Needelman et al. 2018) would contribute no additional soil carbon. This implies no additionality from the global extent of > 25% of mangroves (Sanderman et al. 2018), > 30% of tidal marshes (Maxwell et al. 2023) and > 50% of seagrasses (Fourqurean et al. 2012).

*Zostera sp*. of seagrasses and macrotidal estuarine mangroves are both likely to be subject to large deductions from their soil carbon stores because they typically contain large allochthonous contributions (Krause et al. 2022; Rovai et al. 2018). In regions where accelerating sea‐level rise is a pressure on BCEs, the sites with the greatest potential to keep pace and resist erosion/drowning are likely to be those with the highest sediment availability (Fagherazzi et al. 2020; Saintilan et al. 2020). The BCEs in coastal environments with the greatest sediment availability are also mostly located in estuarine/riverine systems with a high tidal range and high sediment load, which also typically have large proportions of allochthonous carbon (Balke and Friess 2016; Rovai and Twilley 2021). Therefore, in these regions and typologies, excluding allochthonous carbon contributions to BC crediting projects risks excluding the very BCEs which will likely provide the most stable long‐term OC stores, potentially making them financially inviable.

## 7 A Way Forward for the Accounting of Allochthonous OC in BC Crediting Methodologies

We have critiqued the use of the scientific evidence that underpins the treatment of allochthonous carbon in BC methodologies. We also acknowledge the importance of minimising the accounting of any non‐additional allochthonous carbon in BCEs to ensure reliable climate mitigation and genuine emissions abatement. If allochthonous carbon is to be included as avoided emissions in BC crediting projects, it is essential that it be shown to be additional, yet a scientifically rigorous and universally applicable treatment for allochthonous carbon is still to be established.

Removing the requirement of deducting allochthonous carbon would have the benefit of simplifying these methodologies and removing the impetus for projects to either undertake complex and expensive sampling procedures to determine the necessary deductions, or rely on default factors which can be different from empirically measured values (Figure 1), or use regionally specific values which can also be unreliable due to allochthonous contributions varying with geomorphic setting and typology (Rovai et al. 2018; Rovai and Twilley 2021). To mitigate the risk of accounting for any non‐additional allochthonous carbon, where an observational and experimental approach towards calculating the refractory allochthonous carbon contribution is feasible, it should be employed. In cases where direct measurement of the refractory allochthonous contribution is not feasible, we argue that allochthonous carbon should be included in BC crediting projects to avoid the use of deductions based on mathematical conversions which are not supported by adequate empirical evidence.

One potential advance to the issue of accounting for allochthonous carbon is a shift towards projects at a ‘seascape’ scale, for example, an entire estuarine system (Huxham et al. 2018; Krause et al. 2022). Such projects would incorporate both donor and depositional environments and would therefore only be feasible by accounting for allochthonous carbon and measuring the transfer of carbon from one area of the project to another. However, projects at this scale will not be feasible everywhere, and the issue of accounting for allochthonous carbon remains for habitat scale projects.

## 8 Conclusions

The treatment of allochthonous OC in the calculation of additionality in BC crediting projects remains critical to the validity of and confidence in these methodologies. The inconsistencies highlighted between the methodologies reviewed here result in large variability in the amount of carbon deemed additional when applied to an example tidal marsh soil dataset from Mirabito and Chambers (2023) (Table 2, Figure 1).

It is crucial that any BC accounting for climate mitigation is both transparent about its scope (Johannessen and Christian 2023) and follows methodologies which are stringent and backed by accurate scientific data (Van Dam et al. 2024). Whether accounting for allochthonous OC from non‐discounting or discounting approaches, current methodological guidance associated with these approaches can be contradictory and often lacks the scientifically rigorous and universally applicable treatment that is required to account for additionality. We therefore advocate that when an observational and experimental approach does not support the calculation of the refractory allochthonous carbon contribution, no discounting of allochthonous carbon should be applied to BC crediting projects. However, if an observational and experimental approach towards calculating the refractory allochthonous carbon contribution is possible for a BC crediting project, then it should be used as a conservative measure to ensure that any credits issued are genuine and additional.

## Author Contributions

**Alex Houston:** conceptualization, formal analysis, investigation, methodology, project administration, visualization, writing – original draft, writing – review and editing. **Hilary Kennedy:** conceptualization, funding acquisition, writing – review and editing. **William E. N. Austin:** conceptualization, funding acquisition, supervision, writing – review and editing.

## Conflicts of Interest

The authors declare no conflicts of interest.

## Supporting information


Data S1.

## Data Availability

Data sharing is not applicable to this article as no new data were created or analyzed in this study.
