# Supplementary material for: Additionality in Blue Carbon Ecosystems: Recommendations for a Universally Applicable Accounting Methodology
Source: Glob Chang Biol. 2024 Nov 4;30(11):e17559. doi: 10.1111/gcb.17559 (PMC11602935; doi:10.1111/gcb.17559)
Supplement: Supplementary file 1 — Data S1. [file GCB-30-e17559-s001.docx]

**Supporting Information**

**SI Table 1.** Treatment of allochthonous OC by BCE carbon crediting methodologies. Where cells are left blank, there was not sufficient information in the available information online to populate it.

| **Methodology** | **Treatment of Allochthonous OC** | **Reasoning** |
| --- | --- | --- |
| 2013 Supplement to the 2006 IPCC Guidelines for National Greenhouse Gas Inventories: Wetlands Methodological Guidance on Lands with Wet and Drained Soils, and Constructed Wetlands for Wastewater Treatment (IPCC, 2014) | Allochthonous carbon is not mentioned in this documentation. | Pg 176: “The carbon stock is taken as all soil carbon except any refractory (unoxidisable) carbon. In mangrove soils, 4% of the carbon stock is refractory (Annex 4A.4) and this is taken to be representative of the refractory carbon in tidal marshes and seagrass meadows as well.”  Pg 343: “Refractory carbon…Soil carbon that does not get broken down and released as dissolved or gaseous CO2…within the time scale of the inventory.” |
| BlueCAM (Lovelock et al., 2022) | Pg 12: “BlueCAM does not include discounts for allochthonous carbon that is trapped in coastal wetlands and incorporated within soils.” | Pg 12: “While allochthonous carbon has been detected in coastal wetland soils in Australia, contributions of allochthonous carbon were typically small compared to autochthonous sources in saltmarshes and mangroves (Saintilan et al., 2013), but large in some sites for seagrass (Samper-Villarreal et al., 2016).”  Pg 12: “In Australia’s existing accounting framework, organic carbon that is eroded from landscapes and transported to the coast is assumed emitted as CO2, and therefore any portion of this organic carbon trapped in coastal wetlands could be considered an avoided emission (Kelleway et al., 2020)”.  Pg 12: “Future development of BlueCAM could revisit the importance of allochthonous carbon sources and sinks.” |
| VM0033 Methodology for Tidal Wetland and Seagrass Restoration v2.1 (VERRA, 2023) | Pg 38: “A deduction from the estimate of CO_2_ emissions from the SOC pool must be applied to account for the percentage of sequestration resulting from allochthonous soil organic carbon accumulation.”  Pg 38: “Estimation may be made for total or recalcitrant allochthonous carbon.”  Pg 39: “%Calloch may be estimated using either:  1) Published values  2) Field-collected data  3) Modeling.” | Pg 56: “The determination of the deduction for allochthonous carbon is mandatory for the project scenario unless the project proponent is able to demonstrate that the allochthonous carbon would have been returned to the atmosphere in the form of carbon dioxide in the absence of the project.”  Pg 56: “If the organic surface layer exceeds 10 cm, the soil is deemed organic and no deduction is required.” |
| Methodology for Sustainable Management of Mangroves, Public Consultation Draft (Gold Standard, 2024) | Pg 49: “…determine the SOC from autochthonous…and allochthonous…sources. The project developers shall have the  option in terms of using…direct measurement, peer-reviewed data/models or  national/regional default values…when it comes to the accounting of the allochthonous  and autochthonous SOC.” |  |
| The Restoration of California Deltaic and Coastal Wetlands (ACR, 2017) | Pg 90: “In the Project Scenario, net accumulation of allochthonous carbon must be subtracted from the net carbon balance of a wetland unless the project proponent can document that no other entity may claim its GHG emission reductions or removals.”  Pg 89: “Eaq is the deduction to account for allochthonous soil organic carbon.”  Pg 104: “Eaq is the annual net aqueous exchange of carbon in drainage water.” | Pg 68: “Allochthonous carbon may enter the open water area from an outside source and may contribute to carbon accumulation at the site. However, if it represents carbon assimilated by other sinks, the wetland project area does not contribute to its removal from the atmosphere.  Pg 90: “In the Project Scenario, net accumulation of allochthonous carbon must be subtracted from the net carbon balance of a wetland unless the project proponent can document that no other entity may claim its GHG emission reductions or removals (i.e., that no other entity may make an ownership claim to the emission reductions or removals for which credits are sought) and if its storage in the tidal wetland decreases the rate of its decomposition compared to what it would be in the absence of the project (i.e., the case the tidal wetland was not implemented).” |
| Operationalizing blue carbon principles in France: Methodological developments for *Posidonia oceanica* seagrass meadows and institutionalization (Comte et al., 2024). | Allochthonous carbon is not mentioned in this documentation. |  |
| Implementation of blue carbon offset crediting for seagrass meadows, macroalgal beds, and macroalgae farming in Japan (Kuwae et al., 2022). | Allochthonous carbon is not mentioned in this documentation. |  |
| Afforestation and reforestation of degraded mangrove habitats (UNFCC, 2013) | Allochthonous carbon is not mentioned in this documentation. |  |

**Reference List**

ACR. (2017). *The Restoration of California Deltaic and Coastal Wetlands*. https://acrcarbon.org/wp-content/uploads/2023/03/ACR-California-Deltaic-Coastal-Wetlands-v1.1.pdf

Comte, A., Barreyre, J., Monnier, B., de Rafael, R., Boudouresque, C.-F., Pergent, G., & Ruitton, S. (2024). Operationalizing blue carbon principles in France: Methodological developments for *Posidonia oceanica* seagrass meadows and institutionalization. *Marine Pollution Bulletin*, *198*, 115822. https://doi.org/10.1016/j.marpolbul.2023.115822

Gold Standard. (2024). *Methodology for Sustainable Management of Mangroves* [Public Consultation Draft]. https://goldstandard.cdn.prismic.io/goldstandard/ZenKYHUurf2G3MNq_MethodologyforSustainableMangroveManagementforPublicConsultation.pdf

IPCC. (2014). *2013 Supplement to the 2006 IPCC Guidelines for National Greenhouse Gas Inventories: Wetlands Methodological Guidance on Lands with Wet and Drained Soils, and Constructed Wetlands for Wastewater Treatment*. https://www.ipcc-nggip.iges.or.jp/public/wetlands/

Kelleway, J. J., Serrano, O., Baldock, J. A., Burgess, R., Cannard, T., Lavery, P. S., Lovelock, C. E., Macreadie, P. I., Masqué, P., Newnham, M., Saintilan, N., & Steven, A. D. L. (2020). A national approach to greenhouse gas abatement through blue carbon management. *Global Environmental Change*, *63*, 102083. https://doi.org/10.1016/j.gloenvcha.2020.102083

Kuwae, T., Watanabe, A., Yoshihara, S., Suehiro, F., & Sugimura, Y. (2022). Implementation of blue carbon offset crediting for seagrass meadows, macroalgal beds, and macroalgae farming in Japan. *Marine Policy*, *138*, 104996. https://doi.org/10.1016/j.marpol.2022.104996

Lovelock, C. E., Adame, M. F., Bradley, J., Dittmann, S., Hagger, V., Hickey, S. M., Hutley, L. B., Jones, A., Kelleway, J. J., Lavery, P. S., Macreadie, P. I., Maher, D. T., McGinley, S., McGlashan, A., Perry, S., Mosley, L., Rogers, K., & Sippo, J. Z. (2022). An Australian blue carbon method to estimate climate change mitigation benefits of coastal wetland restoration. *Restoration Ecology*, *n/a*(n/a), e13739. https://doi.org/10.1111/rec.13739

Saintilan, N., Rogers, K., Mazumder, D., & Woodroffe, C. (2013). Allochthonous and autochthonous contributions to carbon accumulation and carbon store in southeastern Australian coastal wetlands. *Estuarine, Coastal and Shelf Science*, *128*, 84–92. https://doi.org/10.1016/j.ecss.2013.05.010

Samper-Villarreal, J., Lovelock, C. E., Saunders, M. I., Roelfsema, C., & Mumby, P. J. (2016). Organic carbon in seagrass sediments is influenced by seagrass canopy complexity, turbidity, wave height, and water depth. *Limnology and Oceanography*, *61*(3), 938–952. https://doi.org/10.1002/lno.10262

UNFCC. (2013). *Afforestation and reforestation of degraded mangrove habitats*. https://cdm.unfccc.int/UserManagement/FileStorage/8AE9TYMDSZJP762KF3CL0NWR5HBIUV

VERRA. (2023). *VM0033 Methodology for Tidal Wetland and Seagrass Restoration, v2.1*. https://verra.org/methodologies/vm0033-methodology-for-tidal-wetland-and-seagrass-restoration-v2-1/
